# Analysis of Frailty Syndrome in Men with Metastatic Prostate Cancer: A Scoping Review

**DOI:** 10.3390/jpm13020319

**Published:** 2023-02-13

**Authors:** Mayra Alejandra Mafla-España, María Dolores Torregrosa, Omar Cauli

**Affiliations:** 1 Department of Nursing, University of Valencia, 46010 Valencia, Spain; 2 Frailty Research Organized Group (FROG), University of Valencia, 46010 Valencia, Spain; 3 Medical Oncology Department, Doctor Peset University Hospital, 46017 Valencia, Spain

**Keywords:** docetaxel, chemotherapy, androgen-deprivation therapy, toxicity, geriatric evaluation, quality of life

## Abstract

Most patients with metastatic prostate cancer (mPCa) are older. In addition, current geriatric oncology guidelines suggest that all cancer patients aged over 70 years should undergo a comprehensive geriatric assessment (CGA), with the identification of frailty syndrome being crucial for clinical decisions. Frailty can be associated with lower quality of life (QoL) and interfere with the feasibility or side effects of oncology treatments. Methods: We performed a systematic literature search to evaluate frailty syndrome and associated alterations related to CGA impairment by searching in different academic databases (PubMed, Embase, and Scopus). The identified articles were reviewed according to the Preferred Reporting Items for Systematic Reviews and Meta-Analyses (PRISMA) guidelines. Results: Of the 165 articles consulted, 7 met our inclusion criteria. Analysis of data related to frailty syndrome in patients with mPCa showed a prevalence between 30–70% depending on the tool used. Additionally, frailty was associated with other CGA assessments and QoL evaluation outcomes. In general terms, CGA scores for patients with mPCa were lower than those for patients without metastasis. Furthermore, functional QoL appeared to be worse for patients with metastasis, and global QoL (burden) was more strongly associated with frailty. Conclusion: Frailty syndrome was related to a poorer QoL in patients with mPCa and its evaluation should be considered in clinical decision-making and when choosing the most appropriate active treatment, if any, to increase survival.

## 1. Introduction

Frailty is defined as a state of vulnerability because of poor resolution of homeostasis after a stressful event which subsequently increases the risk of adverse outcomes such as falls, delirium, and disability [1,2]. Frailty is of particular importance in oncology patients. In addition, elderly individuals constitute a sizable proportion of oncology patients and account for approximately 80% of cancer deaths each year [3]. Given that both the cancer itself and oncological therapies can be significant additional stressors that challenge the physiological reserve of patients, the incidence of frailty in older cancer patients is especially high [4]. In fact, more than half of older cancer patients have frailty or prefrailty, and these patients are at an increased risk for postoperative complications, intolerance to chemotherapy, disease progression, and death [5].

Over the past two decades, the concept of frailty has been increasingly recognized as one of the most important problems in healthcare and health outcomes in the elderly. Approximately 10% to 20% of patients over 65 years of age have frailty, and the incidence doubles in those aged over 85 years [5]. Thus, the International Society of Geriatric Oncology (SIOG in its French acronym) has developed guidelines for a comprehensive geriatric assessment (CGA) in elderly cancer patients. These recommendations suggest that all cancer patients aged over 70 years should undergo a comprehensive CGA, which should include assessment of functional status, comorbidity, cognition, mental health status, fatigue, social status, support, nutrition, and the presence of geriatric syndromes [6].

Among the most prevalent cancers, prostate cancer (PCa) is the second most common type with an estimated 1.4 million new cases and 375,000 deaths worldwide per year [7,8,9]. Previous studies have shown that advanced age is the primary risk factor, with more than 75% of all PCas being diagnosed in men over 65 years of age [10,11]. Among patients with PCa, 12–15% had regional disease and only 4–5% had distant disease at the time of diagnosis [12,13]. Notably, a recent population-based study in United States found that the incidence of metastatic PCa (mPCa) is on the rise [3]. The guidelines for the treatment of cancer in general, and PCa specifically, emphasize that decisions in cases of metastatic disease should be based on the physical and mental state of the patient [14]. Considering that many individuals with PCa are elderly, this aspect is especially relevant because aging influences their physical and functional capacity and can influence their response to cancer treatment and its possible side effects.

The objectives of this scoping review were to assess the prevalence of frailty syndrome in patients with mPCa and its relationship with other parameters of the CGA and to compare these results with those from patients with non-metastatic PCa in studies that had compared these two groups of patients.

## 2. Methods

We performed a scoping review to systematically analyze published findings regarding frailty syndrome in PCa, identify the most relevant variables and their associations, and determine points for future research. We searched for articles in relevant electronic databases following the Preferred Reporting Items for Systematic Reviews and Meta-Analyses (PRISMA) guidelines [15].

### 2.1. Literature Search

A literature search was performed in the PubMed, SCOPUS, and Embase academic electronic bibliographic databases. The reference lists of all the selected articles were manually cross-referenced to identify additional articles relevant to the outcomes of this review. The search terms used were “metasta* prostate cancer” and “frailty” and “advanced prostate cancer” and “frailty” in the Title/Summary. The search was designed following a keyword analysis of the available literature obtained from a series of broad searches of the resources listed above.

### 2.2. Inclusion and Exclusion Criteria

The following inclusion criteria were applied: (1) full text in English or Spanish; (2) original research articles; and (3) identification of data related to frailty syndrome in patients with mPCa. To determine which articles to include, we analyzed their titles and abstracts and then retrieved the full texts of those that met the inclusion criteria.

### 2.3. Analysis of Information in the Selected Studies

The results of the database searches were uploaded into a web-based system (Mendeley) which was used to manage the selection process and remove duplicate citations. To determine which studies should be included, we examined their titles and abstracts. The electronic full texts of studies that met the inclusion criteria were then retrieved. We extracted the following data from each article: the number and age of the participants in the mPCa groups, characteristics of the comparison groups (if any), instruments used to evaluate frailty as well as other geriatric assessments, pharmacological treatments, and quality of life (QoL). In addition, the prevalence of frailty syndrome and the relationship between frailty and the main outcome of each study were carefully analyzed.

## 3. Results

**Figure 1:**
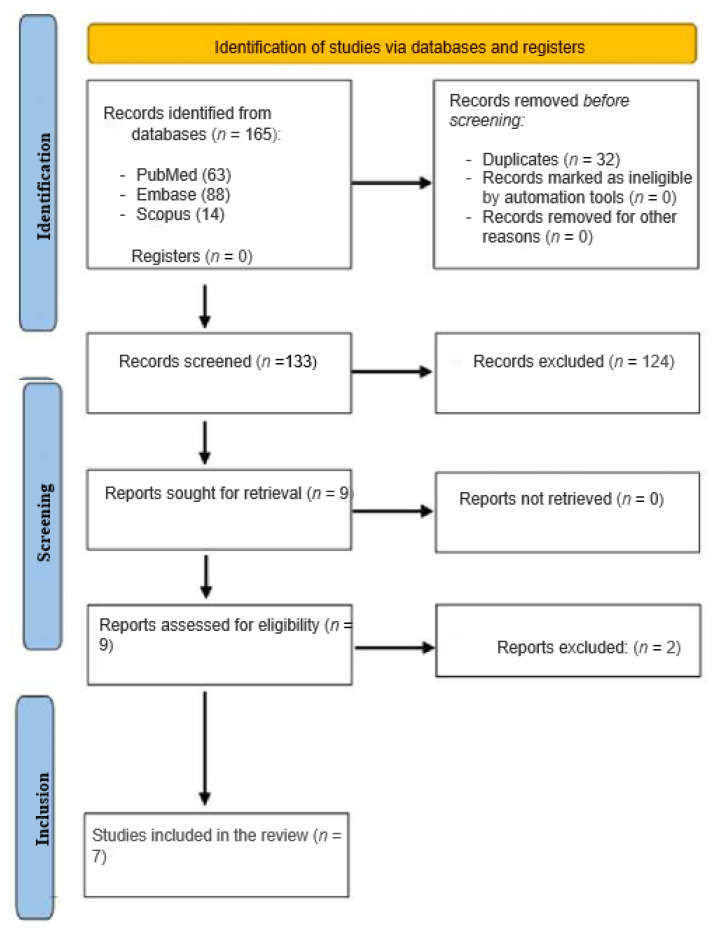
Scoping review workflow.

A total of 165 studies were identified after completing the search process. Excluding duplicate results, we found 9 relevant studies. The full text of each study was then read to decide whether to include or exclude it from this meta-analysis based on the inclusion criteria we had defined. Thus, 7 articles were finally selected [16,17,18,19,20,21,22], given that 2 (out of 9) were abstract proceedings and 124 were excluded because they did not fulfil the criteria. All the participants in the selected studies had mPCa. Figure 1 shows the PRISMA flowchart of the entire review process employed in this present work.

### 3.1. Characteristics of the Studies Analyzed

Of the 7 studies analyzed, 6 were cross-sectional [16,17,18,19,20,21] and 1 was longitudinal [22]. The overall sample size of all selected studies was 276 patients with mPCa, and their mean ages ranged from 70 to 92 years (Table 1). One study focused only on patients with mPCa [19], 4 studies included both metastatic and non-metastatic PCa patients [16,17,21,22], and 2 studies included patients with various types of cancer [18,20].

### 3.2. The Frailty Scales Used

The geriatric screening tool (G8) [16,17], Fried phenotype criteria [17,21,22], CGA [19], Clinical Frailty Scale (CFS) [18], and Modified 5-Factor Frailty Index (mFI-5) [20] instruments were used to assess frailty syndrome. The G8 assesses geriatric domains (food intake, weight loss in the 3 months prior, morbidity, neuropsychological problems, body mass index, use of more than 3 prescription medications per day, general health status compared with peers of the same age, and chronological age) and ranges from 0 to 17 points, with a frailty cut-off score of no more than 14 points [23]. Fried’s frailty criteria functionally assess the frailty phenotype by measuring 5 physical criteria (low grip strength, low energy, slow walking speed, low physical activity, and/or unintentional weight loss); individuals are classified as robust if they do not meet any of these criteria, pre-frail if they meet 1 or 2 criteria, or frail if they meet 3 or more [2]. In turn, the CGA is a multidimensional and multidisciplinary diagnostic and therapeutic tool used to determine the medical, mental, and functional problems of frail older people by assessing the physical, cognitive, psychological, and social functions of daily living [24].

The screening tool used by Pepa et al. [19] was based on CGA scales, e.g., Lawton’s instrumental activities of daily living (IADL) scale; comorbidities with the Cumulative Illness Rating Scale for Geriatrics (CISR-G); emotional state with the Geriatric Depression Scale; risk of isolation with the Lubben scale; and cognitive impairment with the Mini Mental State Exam. Thus, the CGA classifies cancer patients into 4 groups based on their health status, which can help clarify treatment decisions. Patients with frailty are determined by having one or more IADLs, two or more uncontrolled comorbid conditions, or showing severe malnutrition.

The Rockwood Clinical or Modified Frailty Scale instrument is an abbreviated version of Ken Rockwood’s original 70-item frailty index in which disability, cognitive impairment, and the presence of comorbidities were evaluated as predictors of death and institutionalization at 5 years in hospitalized older patients [26]. The severity of frailty syndrome on the Modified Frailty Scale is scored as 4–5 for mild frailty, 6 for moderate frailty, or 7 or more points for severe frailty [27]. The mFI-5 score is calculated based on the presence of 5 comorbidities (congestive heart failure, diabetes mellitus regardless of insulin dependency, chronic obstructive pulmonary disease or pneumonia, partially dependent functional health, and hypertension requiring medication). Each category is assigned 0 points if the comorbidity is absent or 1 point if comorbidity is present [25].

### 3.3. Frailty Analysis between Metastatic and Non-Metastatic Prostate Cancer

Two studies reported a significant difference in the G8 score between the patients without metastases versus those with metastases [16,17]. In this study, the group of patients with metastatic castration-sensitive prostate cancer (mCSPC) had a lower G8 score (<14) than those without metastases (12.5 vs. 14.5, respectively). In other words, 13.8% of the patients with mCSPC were frailer than patients with non-metastatic PCa [16]. In another study, the G8 score was also significantly higher in men without metastases compared to the group with metastasis (14.4 vs. 12.8, respectively), meaning that 11.7% of the patients with metastases were much frailer [17]. In addition, the G8 scores were significantly associated with functional, global, and symptom (burden) QoL scores in the groups with non-metastatic PCa and mCSPC [16].

Together, these results indicated a poorer functional QoL (7.5% lower) in the physical, social, and emotional domains for the group with mPCa versus patients without metastasis, although there were no significant results and differences between the two groups at the cognitive level. In terms of the results for the QLQ-C30, the global QoL was strongly associated with frailty, with 22.7% lower global QoL reported for the group with metastasis. Moreover, there is evidence from studies that used the G8 screening tool with a frailty cut-off of ≤14 in patients with non-metastatic PCa and mPCa demonstrating that patients with frailty had lower G8 scores [17].

### 3.4. The Relationship between Frailty and Other Clinical Variables

One study showed that age was the only sociodemographic variable significantly associated with frailty syndrome, i.e., older patients were frailer than younger patients [22]. Patients with mCSPC had lower scores for QoL (10.6%) based on 100 symptoms (including fatigue, pain, disturbed sleep, nausea/vomiting, appetite loss, constipation, diarrhea, and dyspnea) compared to the group with non-metastatic PCa [28] meaning that they had a poorer QoL [16]. The impact of frailty on chemotherapy treatment (docetaxel as the first-line chemotherapy treatment in mPCa) was significantly associated with frailty (assessed using the CGA) resulting in the early discontinuation of docetaxel (fewer than 3 cycles) [19]. Therefore, 13.6% of patients who had to stop chemotherapy treatment early were healthy, while 60% of them were frailer than those who had completed the treatment plan (>6 cycles) [19]. Similarly, 77% of patients with PCa with spinal metastases who had received their first episode of stereotactic radiotherapy were identified as frail after assessment using the mFI-5 and highlighted the presence of more than 2 of 5 comorbidities (without specifying which criteria were affected). Additionally, this study evaluated the prediction of survival in patients with spinal metastases by measuring the psoas muscle as a hallmark of frailty/sarcopenia, making this metric a simple, objective, and effective way to identify patients with a lower survival risk [20].

## 4. Discussion

Frailty is emerging as one of the most important determinants of health and health outcomes. Both cancer itself and the therapies offered to treat it can be significant additional stressors, challenging patient physiological reserves. In addition, decreased well-being and increased levels of frailty often accompany increasing age, meaning that the incidence of frailty in older cancer patients is especially high. Therefore, frailty can have an influence from a social perspective because it can identify groups of people who need additional medical attention [29]. Our review found a high prevalence of 30–70% frailty syndrome in patients with mPCa, depending on the tools used to evaluate it. Moreover, a consensus about the best tool to measure frailty is still lacking in oncology patients; more than 70 different tools to measure this attribute are available (few of which have been validated), and these range from measuring a single item to assessing more than 90 items. These measures also vary in their intended purpose, with some frailty measures designed as screening tools to risk-stratify patients and others being more formal frailty assessments [30].

Nonetheless, the SIOG recommends the G8 scale [23,31]. Studies using the G8 frailty score threshold of <14 points detected higher levels of frailty in patients with mPCa compared with those without non-metastatic PCa [16,17]. In turn, evaluating frailty syndrome by evaluating the physical phenotype revealed an approximately 30% lower prevalence of frailty [21,22] compared to other tools. However, it should be noted that assessment of the presence of physical frailty is mainly based on signs and symptoms related to decreased energy levels and does not account for specific comorbid conditions or psycho-social variables. Confirming this, the prevalence of frailty among community-dwelling older individuals assessed by the Fried criteria (physical phenotype) was 17.4% worldwide [32]. Indeed, when including the psycho-social variables (as stated in the frailty index based on the accumulation deficits theory of frailty [33]), the prevalence of frailty in the population with mPCa was higher at 50–77% [18,20].

It Is worth noting that a significant association between frailty and health-related QoL (HRQoL) was found in patients with mPCa, with frail patients with mPCa showing a poorer HRQoL compared to those without pCa metastasis. In fact, these results showed that the all-symptom QoL items were significantly worse in the group with mCSPC [16]. This may have been because of the presence of symptoms related to mPCa, with these symptoms perhaps being the same ones that played a key role in producing worse G8 and QoL scores. However, in this study, there were no significant differences between the group with mCSPC and patients without pCa metastases in terms of cognitive function, thereby suggesting that the effect in patients with mPCa was not the result of a general functional decline.

Importantly, the presence of frailty can have an impact on pharmacological treatment in terms of toxicity. In fact, there may be a relationship between frailty and discontinuation of docetaxel treatment (the first-line-chemotherapy agent for treating mPCa) [34] with patients who received fewer than 2 cycles being frailer than those who completed more than 6 cycles [19]. These results have important implications for clinical practice because the recognition of frail patients could help to reduce treatment toxicities and promote the early discontinuation of therapies where appropriate. Indeed, within the framework of the GERICO10-GETUG clinical trial, a preliminary report found that chemotherapy with docetaxel plus prednisone given every 3 weeks was not feasible in frail older (≥75 years) patients with mCRPC [35]. Alternatively, frail patients could be considered for additional therapies to limit the adverse effects of pharmacological treatment, thereby increasing the tolerability and safety of the oncology treatments they are administered [36].

In this sense, accumulating evidence from previous studies has shown that assessment of the frailty status of patients with PCa through comprehensive geriatric assessment tools may be necessary to determine their optimal treatment. These tools have now become an important part of the medical and nursing care of elderly populations [36,37]. Thus, it is important to continually evolve the care of cancer patients, especially in older populations, and, in this context, frailty assessments can inform the best choice and dose of chemotherapy to be administered [30]. Indeed, one of the studies we considered in this work found poorer functional (physical and emotional) QoL scores in patients with mPCa [16].

Thus, it seems that patients with mPCa were more anxious and had more symptoms of depression [38]. Importantly, most patients with advanced cancer have psychological disorders including anxiety and depression resulting from the rapid progression of the disease, intolerance of its clinical symptoms, and other serious complications, which all directly decrease patient QoL and indirectly cause bad health [39]. Furthermore, studies conducted to explore ADT treatment in patients with PCa showed a significant association with increased depression compared to those not treated with ADT [40,41] Nonetheless, more research is required to assess the psychological impact of ADT in patients with mPCa and its relationship with frailty syndrome. This is because depression and frailty syndromes often overlap in frail individuals [42], perhaps even more so in patients with mPCa, which, in turn, can negatively affect their treatment and the disease course [28].

## 5. Conclusions

Despite the wide variety of tools available to measure frailty, the concept of frailty is only beginning to be recognized specifically in cancer patients. It has been identified as a predictor of postoperative complications, chemotherapy intolerance, disease progression, and death [4,30]. In patients with mPCa the rates of frailty are higher compared to individuals with non-mPCa and so these factors should be considered when making clinical decisions. The fact that 20% or more (depending on the tool used) of patients with mPCa assessed were found to be frail also suggested that more aggressive treatments in these patients should be proposed via a multidisciplinary team.

## Figures and Tables

**Table 1:** The mean characteristics of the selected studies.

Reference	Number of Patients with mPCa	Pharmacological Treatment	Instruments Used to Measure Frailty and Other Variables	Prevalence of Frailty in Patients with mPCa
Hamaya et al., 2021 [16]	40	Did not specify.	Frailty was assessed using the G8 screening tool [23]; the European Organization for Research and Treatment of Cancer Quality of Life Questionnaire C30 (QLQ-C30) was also used.	Not reported.
Momota et al., 2020 [17]	96	The G8 score was assessed at the baseline visit prior to treatment. Some patients with mHNPC and every patient with mCRPC was treated with short-term primary ADT or alternative primary ADT antiandrogen therapy, respectively.	Frailty was assessed using the patient’s G8 score [23], from 0 to 17, with a frailty cut-off point of ≤14. The G8 and Fried phenotypes [2] were used.	In the group with mPCa, 70% of the patients were frail (G8 ≤ 14).
Pepa et al., 2017 [19]	24	None of the patients had received chemotherapy and they were all about to start docetaxel. Prior treatment included one to four hormonal manipulations.	Frailty evaluated by the 5 domains of the CGA [24].	79% of the patients were ‘healthy’ while 21% were ‘frail’.
Zakaria et al., 2020 [20]	75	Not specified.	A mFI-5 [25] was used at the time of the first stereotactic body radiation therapy treatment. The mFI-5 score was calculated based on the presence of 5 comorbidities.	77% of the patients had frailty scores > 2.
Navarro-Martinez et al., 2019 [21]	10	All patients were being treated with ADT. Pharmacological treatment with luteinizing hormone-releasing hormone analogues (bicalutamide, leuprorelin, or triptorelin).	The Fried [2] phenotype criteria were used.	10% of the patients were classified as frail (meeting ≥ 3 criteria), 60% were pre-frail (meeting 1 or 2 criteria), and 30% were robust.
Buigues et al., 2020 [22]	7	All the patients were treated with ADT for at least 6 months. Pharmacological treatment with luteinizing hormone-releasing hormone analogues (leuprorelin or triptorelin).	The Fried [2] phenotype criteria were used.	14.3% of the patients were classified as frail (meeting ≥ 3 criteria), 57.1% were pre-frail (meeting 1 or 2 criteria), and 28.5% were robust.
Handforth et al., 2019 [18]	24	All of the patients with PCa received ADT, 20 patients (83%) received corticosteroids, 3 patients (12%) had chemotherapy, and 2 patients (4%) received other systemic treatments.	Patient-self reported frailty score (physician assessment and the Rockwood clinical frailty modified scale) [26].	54.1% of patients were identified as vulnerable or frail.

Abbreviations: mHNPC: metastatic hormone-naïve prostate cancer; mPCa: metastatic prostate cancer; mCRPC: metastatic castration-resistant prostate cancer; QoL: quality of life; ADT: androgen deprivation therapy; G8: geriatric screening tool; CGA: comprehensive geriatric assessment; mFI-5: modified 5-factor frailty index.

## Data Availability

Not applicable.
